# ASO Author Reflections: Long-term Health Outcomes of New Persistent Opioid Use After Gastrointestinal Cancer Surgery

**DOI:** 10.1245/s10434-024-15466-8

**Published:** 2024-05-21

**Authors:** Mujtaba Khalil, Timothy M. Pawlik

**Affiliations:** Department of Surgery, The Ohio State University Wexner Medical Center and James Comprehensive Cancer Center, Columbus, OH USA

## Past

Although the number of opioid prescriptions has decreased in recent years, opioids still pose a healthcare challenge as misuse and overdose rates remain high.^1^ Patients undergoing cancer surgery are more likely to be prescribed higher doses of opioids due to the debilitating postoperative pain resulting from the underlying disease and surgical procedures.^2^ In turn, patients with cancer are at a higher risk of developing opioid use disorders, such as new persistent opioid use (NPOU).^2^ New persistent opioid use occurs when an opioid-naive patient receives an opioid prescription for short-term postoperative pain relief but continues refilling it beyond the expected period for pain resolution.^3^ Despite recent guidelines designed to curb opioid misuse, up to 6% of patients develop NPOU after common surgical procedures.^3^ Importantly, patients undergoing gastrointestinal cancer surgery often receive higher doses of opioids for extended duration and may have an increased risk of developing NPOU.^2^ Nevertheless, the incidence, risk factors, and long-term healthcare outcomes of NPOU among patients undergoing gastrointestinal cancer surgery remain poorly defined. Therefore, the current study sought to evaluate the long-term health outcomes of NPOU among patients undergoing gastrointestinal cancer surgery.

## Present

A total of 15,456 Medicare beneficiaries underwent gastrointestinal cancer surgery (hepatic: n = 288, 1.9%; pancreatic: n = 948, 6.1%; biliary duct: n = 459, 3.0%; colorectal: n = 13,761, 89.0%). Median age was 75 years (IQR 70–81), most patients were female (n = 8435, 54.6%), and had a CCI score ≤ 2 (n = 14,161, 91.6%). The overall incidence of NPOU after gastrointestinal cancer surgery was 6.3% (n = 967). Of note, patients with a history of substance abuse (OR 1.45, 95% CI 1.14–1.84), moderate social vulnerability (OR 1.26, 95% CI 1.06–1.50), higher disease stage (OR 4.42, 95% CI 3.51–5.82), and perioperative opioid use (OR 3.07, 95% CI 2.59–3.63) were at risk for developing NPOU. After controlling for competing risk factors, patients who developed NPOU were more likely to visit a hospital for falls, respiratory events, or pain symptoms (OR 1.45, 95% CI 1.18–1.78). Moreover, patients who developed NPOU had a greater risk of death at 1 year (HR 2.15, 95% CI 1.74–2.66).

## Future

Roughly 1 in 15 patients develop NPOU following gastrointestinal cancer surgery.^4^ NPOU is associated with a greater likelihood of mortality and hospital visits because of falls, respiratory complications, and pain symptoms.^4^ The results of the current study underscore the importance of preoperative risk stratification and comprehensive perioperative pain management plans to prevent NPOU.^4^ In the preoperative setting, patients should be screened for NPOU risk factors, and if opioids are prescribed, there is a need for close monitoring.^5,6^ Additionally, surgical pain may be managed through multimodal analgesia, with a focus on nonopioid medications, such as acetaminophen, nonsteroidal anti-inflammatory drugs, gabapentin, and ketamine.^5^ Moreover, there is a need for opioid stewardship programs that focus on prescription monitoring, patient education, and healthcare provider training.^6^
